# Supplementary material for: COVID-19 Severity and Survival over Time in Patients with Hematologic Malignancies: A Population-Based Registry Study
Source: Cancers (Basel). 2023 Feb 27;15(5):1497. doi: 10.3390/cancers15051497 (PMC10001264; doi:10.3390/cancers15051497)
Supplement: Supplementary file 1 [file cancers-15-01497-s001.zip › cancers-2101980-supplementary.pdf]

RESEARCH

COVID-19 severity and survival over time in patients with hematologic malignancies: a population-based registry study

Supplementary file S1 *(.pdf)*

Supplementary Table S1.

Patient characteristics, hematologic malignancy and therapy received, and clinical severity of COVID-19 and treatment received according to time of diagnosis (early cohort, 1st wave, March-June 2020; later cohort, 2nd/3rd wave, July 2020-February 2021) in the total study population (N = 1166) and all inpatients (N = 896)

Supplementary Figure S1. Survival estimates by HM features.

Survival estimates through day 120 according to (A) type of HM, (B) HM treatment status, (C) type of HM treatment, and (D) transplantation status. *P* values for comparisons between groups were determined by log rank test, with Šidák correction for multiple comparisons; there were no significant differences between groups in any analysis after correction, except for between patients receiving autologous (*P* < .0001) or allogeneic (*P* = .0003) stem cell transplantation and those not receiving stem cell transplantation.

Supplementary Material

Supplementary Table S1. Patient characteristics, hematologic malignancy and therapy received, and clinical severity of COVID-19 and treatment received according to time of diagnosis (early cohort, 1st wave, March-June 2020; later cohort, 2nd/3rd wave, July 2020-February 2021) in the total study population (N = 1166) and all inpatients (N = 896)

	Total study population				Inpatients			
	Total (N = 1166)	Early cohort (n = 769)	Later cohort (n = 397)	Odds ratio (95% CI)	Total (N = 896)	Early cohort (n = 681)	Later cohort (n = 215)	Odds ratio (95% CI)
**Age, y**	**N = 1147**	**n = 767**	**n = 380**		**N = 888**		**n = 207**	
Median (IQR)	71 (59-79)	72 (60-79)	70 (58-78)		72 (63-80)	72 (62-80)	72 (64-80)	
Age 18-59 y, n (%)	287 (25.0)	183 (23.9)	104 (27.4)	reference	177 (19.9)	141 (20.7)	36 (17.4)	reference
Age ≥60 y, n (%)	860 (75.0)	584 (76.1)	276 (72.6)	0.83 (0.63-1.10)	711 (80.1)	540 (79.3)	171 (82.6)	1.24 (0.83-1.86)
**Sex, n (%)**	**N = 1152**	**n = 756**	**n = 396**		**N = 884**	**n = 669**		
Female	454 (39.4)	296 (39.2)	168 (42.4)	reference	348 (39.4)	259 (38.7)	89 (41.4)	reference
Male	698 (60.6)	460 (60.8)	228 (57.6)	1.15 (0.89-1.47)	536 (60.6)	410 (61.3)	126 (58.6)	1.12 (0.82-1.53)
**Comorbidities**	**N = 1166**	**n = 769**	**n = 397**					
Cardiac disease, n (%)	241 (20.7)	160 (20.8)	81 (20.4)	0.98 (0.72-1.32)	198 (22.1)	150 (22.0)	48 (22.3)	1.02 (0.70-1.47)
Respiratory disease, n (%)	171 (14.7)	116 (15.1)	55 (13.9)	0.91 (0.64-1.28)	141 (15.7)	105 (15.4)	36 (16.7)	1.11 (0.73-1.67)
Renal disease, n (%)	127 (10.9)	85 (11.1)	42 (10.6)	0.95 (0.64-1.41)	110 (12.3)	81 (11.9)	29 (13.5)	1.16 (0.73-1.82)
Diabetes, n (%)	200 (17.2)	136 (17.7)	64 (16.1)	0.89 (0.65-1.24)	175 (19.5)	128 (18.8)	47 (21.9)	1.21 (0.83-1.76)
Hypertension, n (%)	453 (38.9)	315 (41.0)	138 (34.8)	0.77 (0.60-0.99)	382 (42.6)	294 (43.2)	88 (40.9)	0.91 (0.67-1.25)
BMI > 35, n (%)	56 (4.8)	38 (4.9)	18 (4.5)	0.91 (0.51-1.62)	49 (5.5)	36 (5.3)	13 (6.1)	1.15 (0.60-2.22)
Number of above 6 comorbidities, n (%)								
0	314 (26.9)	187 (24.3)	127 (32.0)	reference	208 (23.2)	146 (21.4)	62 (28.8)	reference
≥1	852 (73.1)	582 (75.7)	270 (68.0)	0.68 (0.52-0.89)	49 (5.5)	36 (5.3)	13 (6.1)	0.68 (0.48-0.95)
Other comorbidities, n (%)	538 (46.1)	400 (52.0)	138 (34.8)	0.49 (0.38-0.63)	450 (50.2)	372 (54.6)	78 (36.3)	0.47 (0.35-0.65)
**Hematologic malignancy, n (%)**	**N = 1166**	**n = 769**	**n = 397**					
Lymphoid malignancy	839 (72.0)	541 (70.4)	298 (75.1)	reference	626 (69.9)	474 (69.6)	152 (70.7)	reference
Myeloid malignancy	327 (28.0)	228 (29.6)	99 (24.9)	0.49 (0.38-0.63)	270 (30.1)	207 (30.4)	63 (29.3)	0.95 (0.68-1.33)
NHL	325 (27.9)	212 (27.6)	113 (28.5)	reference	249 (27.8)	189 (27.8)	60 (27.9)	reference
MM	283 (24.3)	162 (21.1)	121 (30.5)	1.17 (0.84-1.64)	188 (21.0)	146 (21.4)	42 (19.5)	0.91 (0.58-1.42)
CLL	175 (15.0)	124 (16.1)	51 (12.8)	0.77 (0.52-1.15)	138 (15.4)	103 (15.1)	35 (16.3)	1.07 (0.66-1.73)

	Total study population				Inpatients			
	**Total (N = 1166)**	**Early cohort (n = 769)**	**Later cohort (n = 397)**	**Odds ratio (95% CI)**	**Total (N = 896)**	**Early cohort (n = 681)**	**Later cohort (n = 215)**	**Odds ratio (95% CI)**
HL	50 (4.3)	31 (4.0)	19 (4.8)	1.15 (0.62-2.13)	34 (3.8)	27 (4.0)	7 (3.3)	0.81 (0.34-1.97)
ALL	26 (2.2)	12 (1.6)	14 (3.5)	2.19 (0.98-0.89)	17 (1.9)	9 (1.3)	8 (3.7)	2.8 (1.04-7.58)
MDS	115 (9.9)	81 (10.5)	34 (8.6)	0.79 (0.50-1.25)	101 (11.3)	78 (11.5)	23 (10.7)	0.93 (0.54-1.61)
AML	92 (7.9)	63 (8.2)	29 (7.3)	0.86 (0.53-1.42)	76 (8.5)	56 (8.29	20 (9.3)	1.13 (0.63-2.02)
CML	33 (2.8)	17 (2.2)	16 (4.0)	1.77 (0.86-3.63)	20 (2.2)	13 (1.9)	7 (3.2)	1.70 (0.65-4.45)
Ph-MPN	87 (7.5)	67 (8.7)	20 (5.0)	0.56 (0.32-0.97)	73 (8.2)	60 (8.8)	13 (6.1)	0.68 (0.35-1.33)
**Stem cell transplantation,* n (%)**	**N = 1127**	**n = 746**	**n = 381**		**N = 868**	**n = 660**	**n = 208**	
Autologous	100 (8.9)	58 (7.8)	42 (11.0)	1.53 (1.01-2.33)	64 (7.4)	44 (6.7)	20 (9.6)	1.57 (0.90-2.73)
Allogeneic	56 (5.0)	29 (3.9)	27 (7.1)	1.97 (1.15-3.38)	43 (5.0)	26 (3.9)	17 (8.2)	2.26 (1.20-4.26)
No	971 (86.2)	659 (88.3)	312 (81.9)	reference	761 (87.7)	590 (89.4)	171 (82.2)	reference
**Cancer therapy, within 30 d,† n (%)**	**N = 1162**	**n = 766**	**n = 396**		**N = 894**	**n = 679**		
No active therapy	483 (41.6)	323 (42.2)	160 (40.4)	reference	362 (40.5)	278 (40.9)	84 (39.1)	reference
Active therapy	679 (58.4)	443 (57.8)	236 (59.6)	1.07 (0.84-1.37)	532 (59.5)	401 (59.1)	131 (60.9)	1.08 (0.79-1.47)
Conventional chemotherapy	260 (22.4)	151 (19.6)	109 (27.5)	1.46 (1.07-1.99)	197 (22.0)	140 (20.6)	57 (26.5)	1.35 (0.91-2.00)
Low-intensity chemotherapy	71 (6.1)	53 (6.9)	18 (4.5)	0.69 (0.39-1.21)	58 (6.5)	46 (6.8)	12 (5.6)	0.86 (0.44-1.71)
Molecular targeted therapy	130 (11.2)	89 (11.6)	41 (10.3)	0.93 (0.61-1.41)	106 (11.9)	80 (11.8)	26 (12.1)	1.08 (0.65-1.78)
Immunotherapy, mAb only	56 (4.8)	43 (5.6)	13 (3.3)	0.61 (0.32-1.17)	45 (5.0)	37 (5.5)	8 (3.7)	0.72 (0.32-1.60)
Immunomodulator drugs	71 (6.1)	44 (5.7)	27 (6.8)	1.24 (0.74-2.07)	51 (5.7)	39 (5.7)	12 (5.6)	1.02 (0.51-2.03)
Hypomethylating agents	49 (4.2)	33 (4.3)	16 (4.0)	0.98 (0.52-1.83)	41 (4.6)	30 (4.4)	11 (5.1)	1.21 (0.58-2.53)
Supportive therapy	31 (2.7)	22 (2.9)	9 (2.3)	0.83 (0.37-1.84)	26 (2.9)	21 (3.1)	5 (2.3)	0.79 (0.29-2.15)
Active, not detailed	11 (0.9)	8 (1.0)	3 (0.8)	0.76 (0.20-2.89)	8 (1.5)	8 (1.2)	0	–
**Pharmacologic therapies for COVID-19, n (%)**	**N = 1166**	**n = 769**	**n = 397**					
(Hydroxy)chloroquine	644 (55.2)	643 (83.6)	1 (0.3)	0.00 (0.00-0.00)	602 (67.2)	602 (67.2)	0	0.00
Azithromycin	381 (32.7)	348 (45.3)	33 (8.3)	0.11 (0.07-0.16)	355 (39.6)	327 (48.0)	28 (13.0)	0.16 (0.11-0.25)
Lopinavir/darunavir	409 (35.1)	409 (53.2)	0	0.00 (0.00-0.00)	392 (43.8)	392 (43.8)	0	0.00
Remdesivir	77 (6.6)	22 (2.9)	55 (13.9)	5.46 (3.28-9.10)	77 (8.6)	22 (3.2)	55 (25.6)	10.3 (6.1-17.4)
Tocilizumab	175 (15.0)	134 (17.4)	41 (10.3)	0.55 (0.38-0.79)	175 (19.5)	134 (19.7)	41 (19.1)	0.96 (0.65-1.42)
Corticosteroids	626 (53.7)	392 (51.0)	234 (58.9)	1.38 (1.08-1.76)	583 (65.1)	386 (56.7)	197 (91.6)	8.36 (5.0-13.9)

	Total study population				Inpatients			
	**Total (N = 1166)**	**Early cohort (n = 769)**	**Later cohort (n = 397)**	**Odds ratio (95% CI)**	**Total (N = 896)**	**Early cohort (n = 681)**	**Later cohort (n = 215)**	**Odds ratio (95% CI)**
**Requirement for oxygen support during COVID-19 treatment, n (%)**	**N = 1162**	**n = 766**	**n = 396**		**N = 892**	**n = 678**	**n = 214**	
No	333 (28.7)	161 (21.0)	172 (43.4)	reference	110 (12.3)	84 (12.4)	26 (12.2)	reference
Low-flow oxygen support	566 (48.7)	442 (57.7)	124 (31.3)	0.26 (0.20-0.35)	519 (58.2)	431 (63.6)	88 (41.1)	0.66 (0.40-1.08)
High-flow oxygen support or mechanical ventilation	263 (22.6)	163 (21.3)	100 (25.3)	0.57 (0.41-0.80)	263 (29.5)	163 (24.0)	100 (46.7)	1.98 (1.20-3.29)
**Clinical severity of COVID-19, n (%)**	**N = 1131**	**n = 760**	**n = 371**		**N = 886**	**n = 677**	**n = 209**	
Mild	254 (22.5)	114 (15.0)	140 (37.7)	2.77 (1.93-3.99)	68 (7.7)	55 (8.1)	13 (6.2)	0.84 (0.42-1.67)
Moderate	254 (22.5)	176 (23.2)	78 (21.0)	reference	195 (22.0)	152 (22.5)	43 (20.6)	reference
Severe	408 (36.1)	319 (42.0)	89 (24.0)	0.63 (0.44-0.90)	408 (46.1)	319 (47.1)	89 (42.6)	0.99 (0.65-1.49)
Critical	215 (19.0)	151 (19.9)	64 (17.3)	0.96 (0.64-1.42)	215 (24.3)	151 (22.3)	64 (30.6)	1.50 (0.96-2.34)
**Care setting of COVID-19 treatment, n (%)**	**N = 1166**	**n = 769**	**n = 397**					
Outpatient	270 (23.2)	88 (11.4)	182 (45.8)	6.55 (4.87-8.82)	–	–	–	–
Inpatient	896 (76.8)	681 (88.6)	215 (54.2)	reference	–	–	–	reference
Intensive care unit	273 (23.4)	170 (22.1)	103 (25.9)	1.25 (0.94-1.66)	273 (30.6)	170 (25.1)	103 (48.1)	2.77 (2.01-3.82)

*Patients who had ever received an autologous or allogeneic stem cell transplant. †Cancer therapy received within 30 days before COVID-19 diagnosis date.

ALL, acute lymphoid leukemia; AML, acute myeloid leukemia; BMI, body mass index; CLL, chronic lymphocytic leukemia; CML, chronic myeloid leukemia; COVID-19, coronavirus disease 2019; HL, Hodgkin lymphoma; IQR, interquartile range; MDS, myelodysplastic syndrome; mAb, monoclonal antibody; MM, multiple myeloma; NHL, non-Hodgkin lymphoma; Ph-MPN, Philadelphia chromosome-negative myeloproliferative neoplasm.

Supplementary Figure S1. Survival estimates by HM features.

Survival estimates through day 120 according to (A) type of HM, (B) HM treatment status, (C) type of HM treatment, and (D) transplantation status. *P* values for comparisons between groups were determined by log rank test, with Šidák correction for multiple comparisons; there were no significant differences between groups in any analysis after correction, except for between patients receiving autologous (*P* < .0001) or allogeneic (*P* = .0003) stem cell transplantation and those not receiving stem cell transplantation.

A

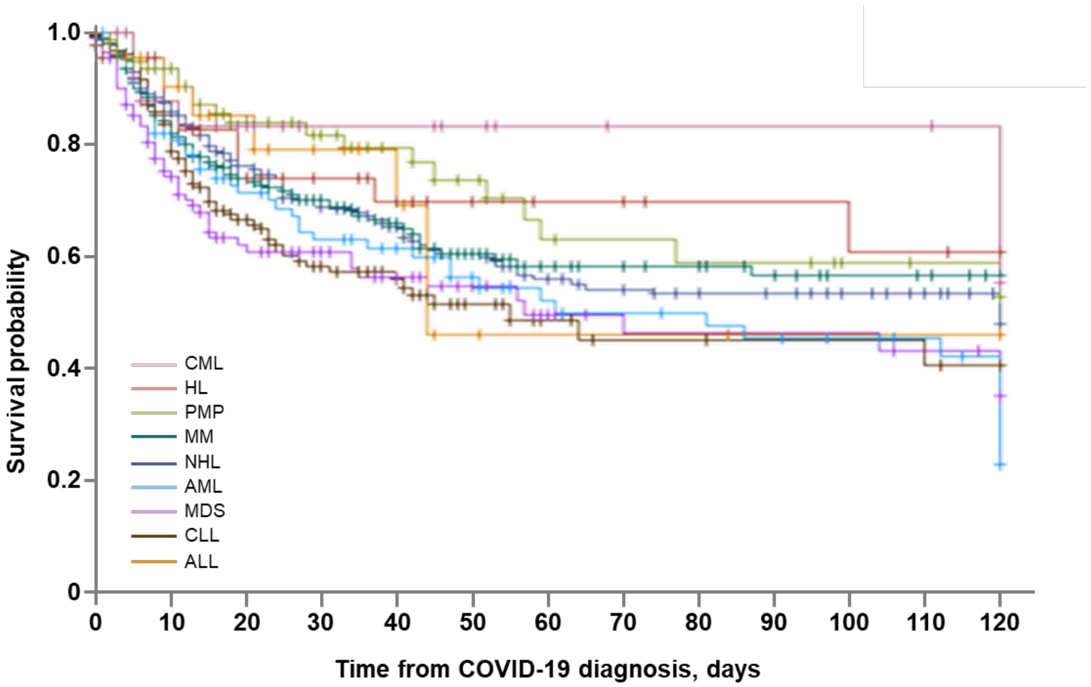

B

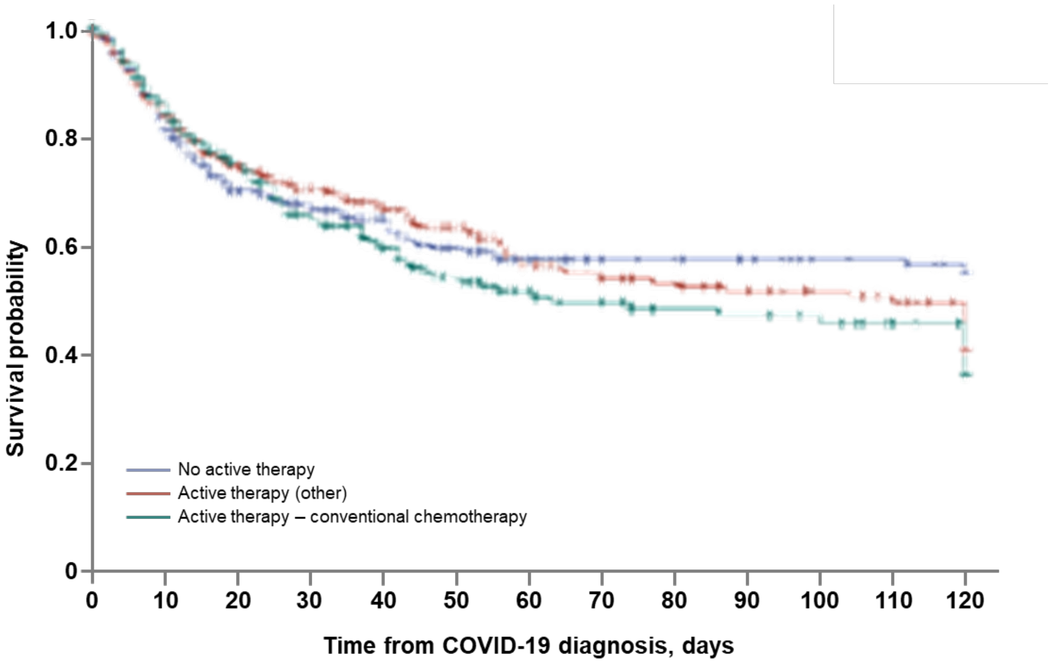

C

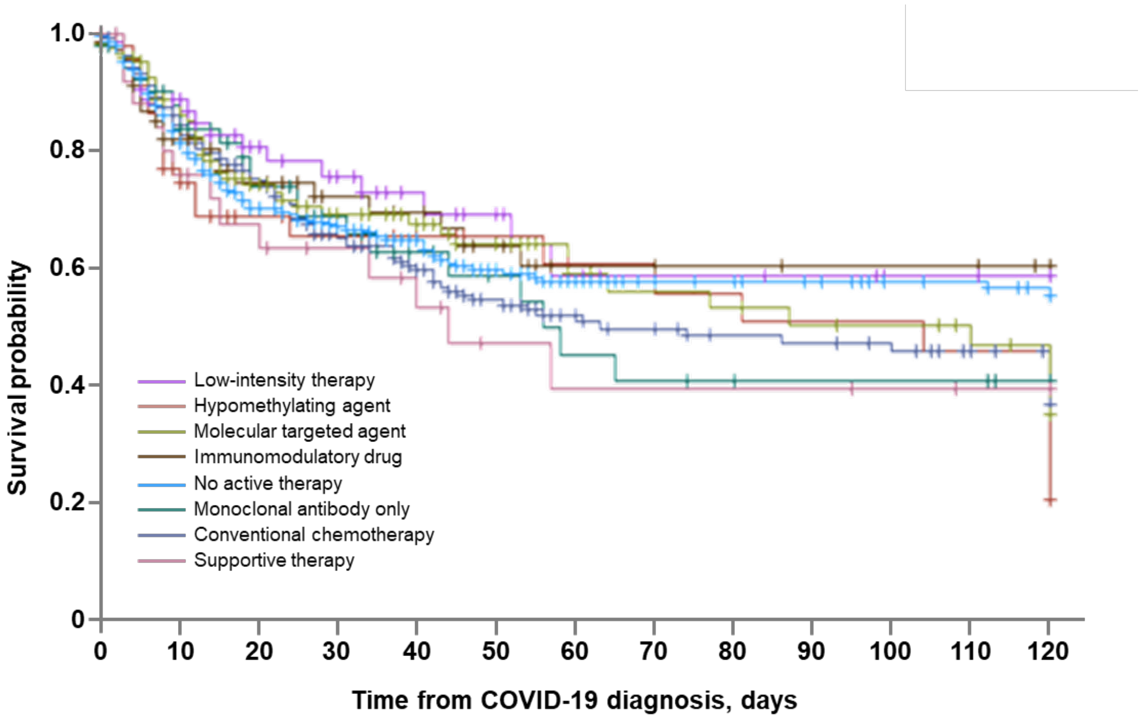
Survival probability
1.0
0.8
0.6
0.4
0.2
0
0 10 20 30 40 50 60 70 80 90 100 110 120
Time from COVID-19 diagnosis, days
Low-intensity therapy
Hypomethylating agent
Molecular targeted agent
Immunomodulatory drug
No active therapy
Monoclonal antibody only
Conventional chemotherapy
Supportive therapy

D

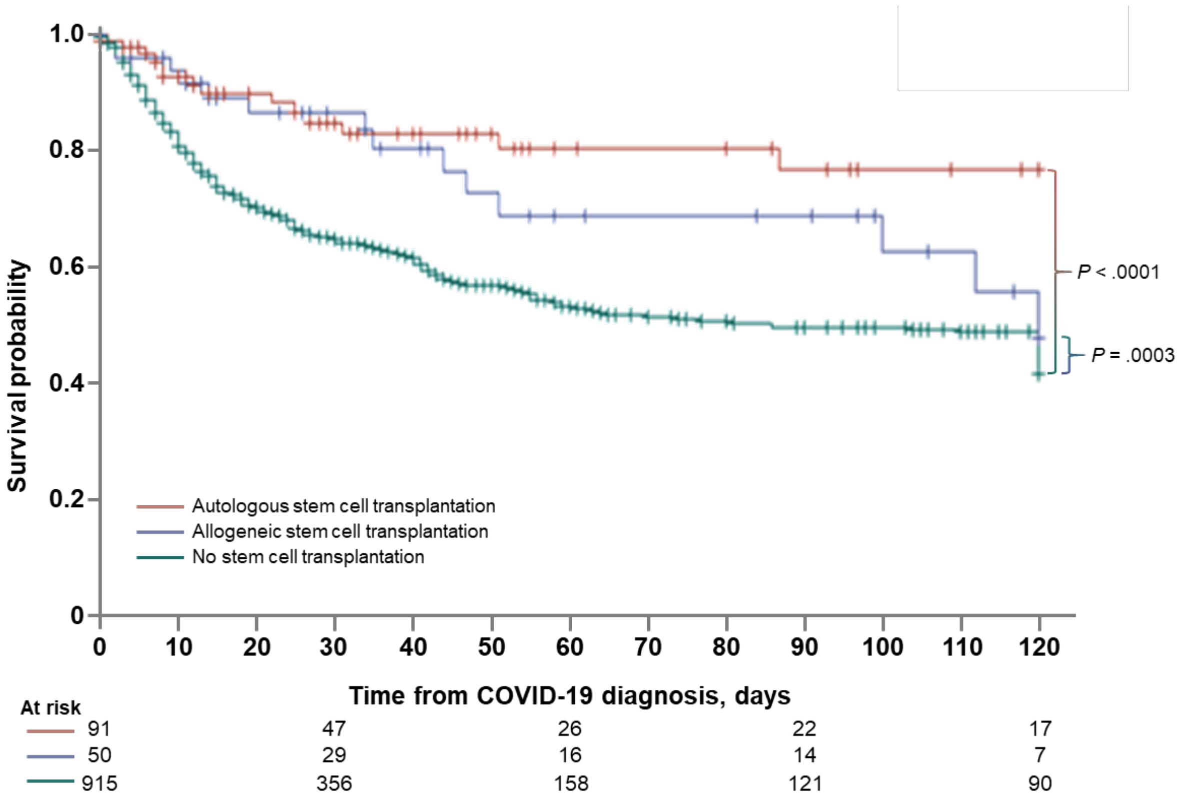
Survival probability
1.0
0.8
0.6
0.4
0.2
0
0 10 20 30 40 50 60 70 80 90 100 110 120
Time from COVID-19 diagnosis, days
P < .0001
P = .0003
Autologous stem cell transplantation
Allogeneic stem cell transplantation
No stem cell transplantation
At risk
91 47 26 22 17
50 29 16 14 7
915 356 158 121 90
